# Oxidative Stability of a New Peanut Butter Bite Product

**DOI:** 10.1155/2021/5528315

**Published:** 2021-06-19

**Authors:** Pranav Kaushik Pidatala, Danielle Bellmer, William McGlynn

**Affiliations:** ^1^Nutradried Food Company, LLC, 6920 Salashan Pkwy Ste #D111, Ferndale, WA 98248, USA; ^2^Robert M. Kerr Food and Agricultural Products Center, Oklahoma State University, Stillwater, OK 74078, USA

## Abstract

Peanut butter continues to be a mainstay in the American diet, but in its current form, peanut butter lacks the convenience of other foods. A peanut butter bite snack food has been developed that is individually wrapped, high in protein, and made mostly from peanut butter. The target market for the product is the active, health-conscious segment of the population that wants a high-protein peanut butter snack that is easy to pack, carry, and eat. The objective of the current study was to evaluate the shelf life of peanut butter bites under different storage and packaging conditions and specifically to monitor oxidative stability of the samples over time. Peanut butter bite samples were prepared with three different levels of added antioxidant (vitamin E). Products were sealed in two different types of packaging (metallized polyethylene and plastic polyethylene) and were stored at two different temperatures to determine the rate of deterioration of the product under various conditions. Oxidative stability was evaluated using two different analytical methods (peroxide value and TBARS assay) to evaluate primary and secondary oxidation products over a six month time period. All treatments were conducted in triplicate. Results show that higher levels of vitamin E resulted in greater stability. As expected, oxidation proceeded more quickly under higher temperature storage conditions. A shelf stable individually wrapped peanut butter snack product may be appealing to a large audience and could result in an increase in the consumption of peanuts.

## 1. Introduction

Peanuts are a valuable component in the human diet, and in 2017, production of peanuts worldwide was about 45 million metric tons [1]. In the United States, about 60% of the peanuts grown are made into peanut butter, amounting to more than 1.8 billion dollars in peanut butter sales [2]. Peanut butter is both nutritious and economical and is popularly used as a snack option and as a sandwich spread. Due to its high protein and nutrient content, it has been used in diet programs to treat malnutrition in African children [3]. In its current form, however, peanut butter lacks the convenience of other snack foods. A peanut butter snack food, which will be called peanut butter bites, has been developed that is individually wrapped, high in protein, great tasting, and made mostly from peanut butter. Formulation of peanut butter bites involves application of an enzyme technology in the presence of additives, which converts semisolid peanut butter into solid chunks that are easily peeled from the wrapper [4].

Peanut butter is a high-lipid food product, so it is highly prone to oxidative rancidity. The most common approach to control oxidation is by adding antioxidants into food products [5]. Vitamin E (alpha tocopherol) is used as an antioxidant in many different applications and has been shown to successfully slow the oxidation process in numerous food products including butter, fish fillets, energy bars, and meat products [6–8].

Packaging material and storage temperature have also been shown to be key factors in preserving the oxidative stability of peanut products [9, 10]. The oxygen and moisture permeability of the package is important, as it affects the amount of oxygen contacting the oxidizable substrates. In addition, exposure to light can also accelerate the oxidation process, and the use of metallized film with a light barrier can increase the shelf life of high-fat products [11]. Oxidation has also been shown to occur much more rapidly at elevated temperatures [9, 12].

Oxidative rancidity in high-fat products is most commonly evaluated using lipid oxidation measurements. The peroxide value (PV) is a measure of the peroxides and hydroperoxides formed during the initial stages of oxidation and has been used to evaluate numerous oilseed products including peanut butter, shea butter, and sunflower kernels [13–16]. The thiobarbituric acid reactive substance (TBARS) assay evaluates rancidity caused by secondary oxidation compounds in high-lipid food systems and measures the aldehydes formed as hydroperoxides break down. These two measurements provide a good indication of oxidative stability of high-fat food products.

The goal of the present study was to evaluate the rate of oxidation of a newly developed peanut butter bite product with varying levels of antioxidant under different storage conditions. A six-month shelf life study was conducted with three different antioxidant levels, two different packaging materials, and two different storage temperatures.

## 2. Materials and Methods

### 2.1. Peanut Butter Bite Preparation

Peanut butter bites were formulated using Jif™ peanut butter. Each sample weighed 12.5 grams and contained peanut butter as the major ingredient along with binding, sweetening, and emulsifying agents. The specific composition of the peanut butter bites is shown in Table 1. Preparation of peanut butter bites involved melting creamy peanut butter (Jif) at 50°C using a water bath (Precision, Reciprocal Shaking Bath Model 50). Pea protein (Growing Naturals), peanut flour (Protein Plus), cacao butter (Navitas), and Alphadim PBK mono-di-glycerides (Corbion) were added to the peanut butter and mixed. This mixture was kept at 50°C. Brown sugar (Great Value) and Kelcogel gellan gum (Modernist Pantry) were added to water in a glass beaker and heated to 90°C using a water bath (Thermo, Microprocessor-controlled Bath 280 Series) and mixed thoroughly. The gel solution was added to the peanut butter mixture and mixed rigorously. After cooling the mixture to 45°C, ACTIVA® Transglutaminase GS enzyme (Ajinomoto) was added to the mixture and mixed. The mixture was immediately scraped into molds covered with Saran™ to form peanut butter bites. After cooling for 30-45 min, the peanut butter bites were packed tightly in Saran™ wrap.

For shelf life studies, 3 different formulations of peanut butter bites were prepared, including a control (no vitamin E), 0.01 gm (200 ppm) of vitamin E, and 0.025 gm (500 ppm) of vitamin E. The formulation used for all peanut butter bite samples was identical except for the levels of vitamin E. From the 50 g formula weight, 4 bites were prepared. Each Saran™-wrapped snack bite was packaged in one of two different packaging materials: 2 mil transparent polyethylene (Uline, IL, S-947) and 2.5 mil metallized polyester and polyethylene bonded film (Uline, IL, S-11661). The packaging materials were selected based on common materials used for packing food products, specifically to pack oxidation-sensitive food products. The Saran™ was used as a primary packing material to hold the integral structure of the bite, and the metallized and/or transparent polyethylene bags served as a secondary packing material. Each package was sealed using an impulse heat sealer (Pack Secure, OH, MP-12C). Oxygen and moisture permeability properties of the packaging materials are shown in Table 2.

### 2.2. Experimental Design

The design of the experiment was based on evaluation of oxidative rancidity using two different oxidation measurements for primary and secondary oxidations (peroxide value and TBARS, described below) of the peanut butter bites stored at two different temperatures, three antioxidant levels, and two different packaging materials. The two temperatures tested were room temperature (25°C ± 5°C) and oven temperature (50°C ± 1°C), which was chosen based on the most probable upper temperature limit during transportation and storage. The three antioxidant levels were 0, 200, and 500 ppm of vitamin E, and the two different packaging materials were a plastic and a metallized package, as described above. Experiments were carried out over 6 months of storage time, with 10 observations in total. The first measurements were taken on the formulation day, the next 5 observations were taken on a weekly basis, and the last 4 observations were taken on a monthly basis. There were three replications of each unique sample.

In total, for every observation day, for either the PV or the TBARS assay, there were 2 × 2 × 3 × 3 = 36 samples. Testing was done at 10 different times, so there were 360 samples tested for peroxide value assay and 360 samples for TBARS assay. All the samples were given a unique number immediately after the formulation. Statistically, this was an experimental project with a 2 × 2 × 3 × 10 factorial treatment structure in a completely randomized design (CRD) with 3 replications at each combination of storage, antioxidant level, and packaging material tested at 10 time periods for both peroxide value and TBARS assays. There were 4 main source factors: temperature, packaging, antioxidant concentration, and time interval; 6 two-way interactions; 4 three-way interactions; and 1 four-way interaction. The oxidation data was analyzed using SAS Institute (Version 9.4, Cary, NC). The statistical analysis methods used were ANOVA, *F*-test, least square means, and difference in least square means. All estimations were done at 95% confidence intervals (type 3 tests of fixed effects).

### 2.3. Peroxide Value Assay

When lipids are oxidized, hydroperoxides are formed that are unstable in nature. These are primary oxidation compounds formed during the early stages of rancidity. The most commonly used quantitative assay for hydroperoxide detection is the peroxide value (PV) assay. It measures the amount of peroxide formed and is expressed in units of milliequivalents of peroxide per kilogram of fat, which is a direct measure of the amount of fat that has been oxidized. The PV assay was conducted according to the AOAC official method 965.33. For PV analysis, 5 g of peanut butter bite sample was homogenized in 30 ml 3 : 2 glacial acetic acid and chloroform mixture. To this mixture, 0.5 ml of saturated potassium iodide solution was added. Peroxides formed in the peanut butter reacted with liberated iodine, which was chemically titrated against sodium thiosulfate solution using starch as an indicator. The titration value gave an estimate of the peroxide value.

The peroxide value (PV) was calculated as follows:

(1) $$\begin{array}{c} \text{PV}=\left(S-B\right)*N*\frac{1000}{\text{sample weight}} \end{array}$$

where *S* is the titration value of the sample (ml), *B* is the titration value of the blank sample (ml), *N* is the normality of sodium thiosulfate solution (0.1 N), and sample weight = 5 grams.

### 2.4. TBARS Assay

The thiobarbituric acid reactive substance (TBARS) assay is an analytical method that quantifies rancidity caused by secondary oxidation compounds in high-lipid food systems. This method gives a measure of the aldehydes present in a food product. After a period of time, unstable primary oxidation compounds (hydroperoxides) break down and aldehydes or ketones are formed. Malondialdehyde (MDA) reacts with thiobarbituric acid (TBA) and trichloroacetic acid (TCA) to form a pinkish-red colored complex. The absorbance of this oxidized chromogen, MDA, is measured at 531 nm using a spectrophotometer. The AOCS Cd 19-90 method [17] was used for the TBARS assay, where 5 g of peanut butter bite sample was dissolved in 15 ml of distilled water and homogenized thoroughly. To prepare TCA solution, 20 g of trichloroacetic acid (TCA) was dissolved in 100 ml distilled water and mixed thoroughly. This solution was kept in a closed glass bottle and stored under a fume hood. To prepare the TCA-TBA reagent, 0.6 g of thiobarbituric acid (TBA) was dissolved in 120 ml of TCA solution and mixed thoroughly. This solution was kept in a cool place in a closed glass bottle. 2 ml of the TCA-TBA reagent was added to 1 ml of peanut butter homogenate and 50 μl of butylated hydroxyanisole was added immediately to avoid further oxidation during the sample analysis. The solution was heated for 15 minutes in a boiling water bath and then cooled for 10 minutes in ice water. The mixture was centrifuged at 2000 G for 10 min (Fisher Scientific Model 225 centrifuge). Absorbance was measured using a spectrophotometer at 531 nm wavelength. A standard curve was developed to calculate the concentration of MDA from absorbance values.

## 3. Results and Discussion

**Figure 1:**
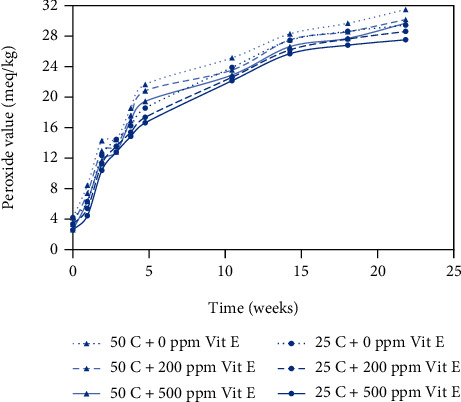
Peroxide values for peanut butter bite samples in plastic packaging material stored at 25°C and 50°C. Values shown are the average of three replicates.

**Figure 2:**
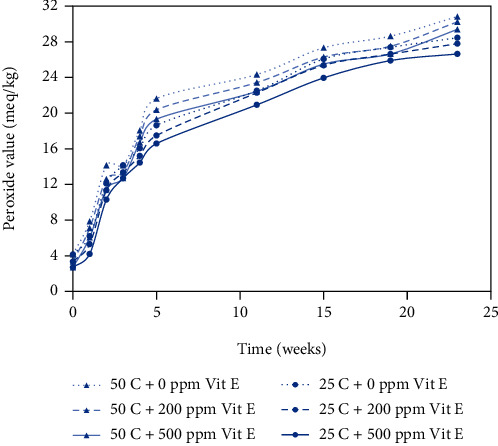
Peroxide values for peanut butter bite samples in metallized packaging material stored at 25°C and 50°C. Values shown are the average of three replicates.

Peanut butter has a high unsaturated fatty acid content, which makes it susceptible to oxidation, and the peroxide value is a measure of the peroxides and hydroperoxides formed during the initial stages of oxidation. The results obtained from the peroxide value assay are shown in Figures 1 and 2 for plastic and metallized packaging materials, respectively. During the 6-month storage period, the peroxide values ranged from 2.6 to 31.51 meq/kg, and all the curves followed a similar trend. The three lower curves show the peroxide value results for peanut butter bite samples stored at 25°C, and the three upper curves show the peroxide values for peanut butter bite samples stored at 50°C. Samples stored at 50°C had consistently higher peroxide values than the samples stored at 25°C. Samples with different levels of antioxidant concentration (200 ppm and 500 ppm) exhibited lower peroxide values than the control samples (0 ppm), and the samples with 500 ppm antioxidant showed the lowest peroxide values, indicating the greatest amount of oxidative stability.

**Figure 3:**
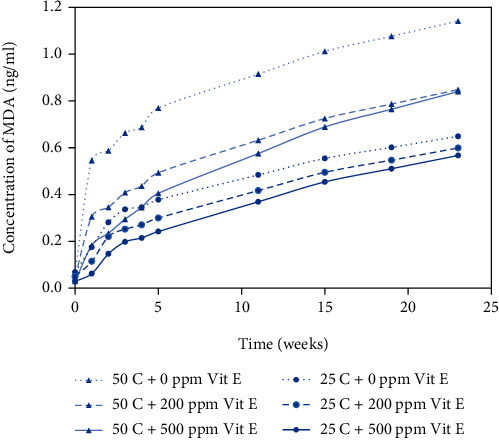
TBARS values for peanut butter bite samples in plastic packaging material stored at 25°C and 50°C. Values shown are the average of three replicates.

**Figure 4:**
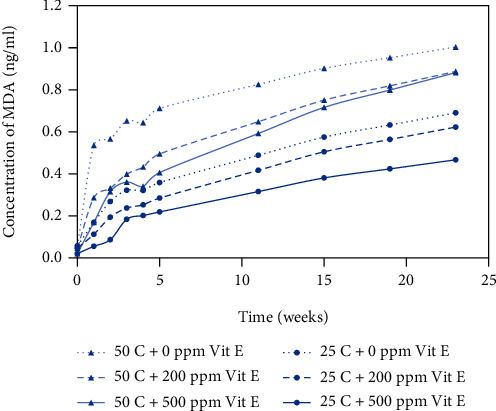
TBARS values for peanut butter bite samples in metallized packaging material stored at 25°C and 50°C. Values shown are the average of three replicates.

The results obtained from the TBARS assay are shown in Figures 3 and 4 for plastic and metallized packaging materials, respectively. TBARS values are an indirect measure of the aldehydes formed during lipid oxidation, and aldehydes are secondary or tertiary byproducts of the fatty acids in the product. TBARS values ranged from 0.02 to 1.16 ng/ml depending on the packaging material and storage conditions. The three lower curves show the TBARS values for peanut butter bite samples stored at 25°C, and the three upper curves show the TBARS values for peanut butter bite samples stored at 50°C. Samples stored at 50°C had significantly higher TBARS values than the samples stored at 25°C. Samples with different levels of antioxidant concentration (200 ppm and 500 ppm) exhibited lower TBARS values than the control samples (0 ppm). The samples with 500 ppm vitamin E showed the lowest TBARS values, indicating the greatest amount of oxidative stability among the different treatment levels.

In Figures 1–4, the samples with 0 ppm vitamin E stored at 50°C exhibited the highest oxidation product values among the six treatments whereas samples with 500 ppm vitamin E stored at 25°C exhibited the lowest values.

### 3.1. Comparison at Specific Time Points

**Figure 5:**
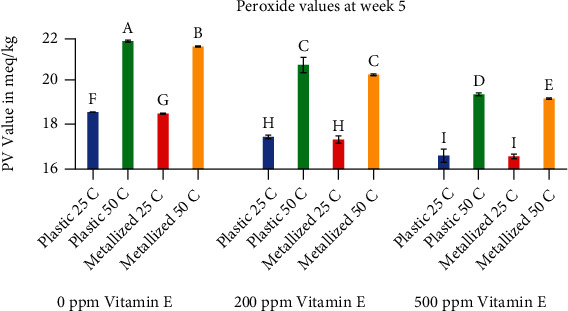
Comparison of peroxide values at week 5. Values shown are the average of three replicates. Error bars represent standard deviation. Values marked with the same letter are not significantly different (*α* = 0.05).

**Figure 6:**
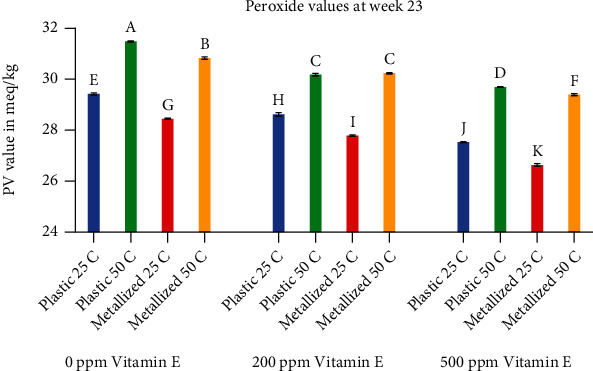
Comparison of peroxide values at week 23. Values shown are the average of three replicates. Error bars represent standard deviation. Values marked with the same letter are not significantly different (*α* = 0.05).

**Figure 7:**
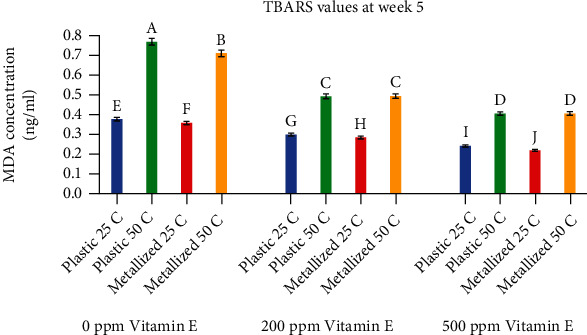
Comparison of TBARS values at week 5. Values shown are the average of three replicates. Error bars represent standard deviation. Values marked with the same letter are not significantly different (*α* = .05).

**Figure 8:**
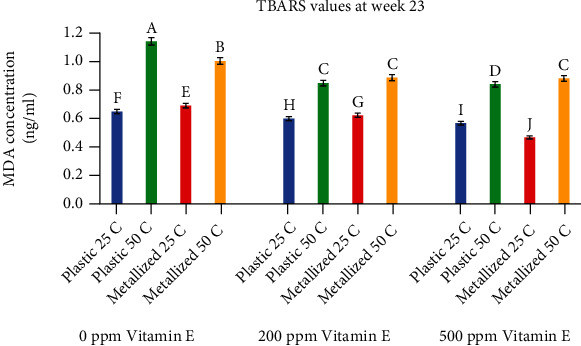
Comparison of TBARS values at week 23. Values shown are the average of three replicates. Error bars represent Standard Deviation. Values marked with the same letter are not significantly different (*α* = 0.05).

In order to more easily compare the twelve different treatments, snapshots of the PV and TBARS values at two different time points (week 5 and week 23) are shown in Figures 5–8.  Figure 5 shows peroxide values for all treatments at week 5. It can be observed from the figure that temperature had the biggest effect on peroxide values, antioxidant concentration had the second largest effect, and packaging had the smallest effect on the peroxide values. Most treatments were significantly different from each other, with the exception of a few treatments where packaging was not significantly different. Peroxide values for samples stored in plastic and metallized packaging materials ranged from 16.5 to 18.8 meq/kg for samples stored at 25°C. Samples stored at 50°C exhibited peroxide values between 19.2 and 21.9 meq/kg for both packaging materials. The samples with 500 ppm vitamin E at 25°C had the lowest peroxide values, but the two different packaging materials were not significantly different at that point. The samples with 0 ppm vitamin E had significantly different peroxide values with respect to temperature and packaging. The samples with 200 ppm vitamin E were significantly different with respect to temperature, but the PV values for samples at 50°C and 25°C did not show significant differences between the plastic and metallized packaging materials. Again, temperature and antioxidant concentration were significantly different in every case, but packaging was not significantly different for several treatments.

Figure 6 shows peroxide values for all treatments at week 23. Again, it is clear from the figure that temperature had the biggest effect on PV values. All treatments were significantly different from each other, with the exception of one pair. The values for samples with 200 ppm vitamin E at 50°C were not significantly different between the plastic and metallized packaging materials.

Figure 7 shows TBARS values for all treatments at week 5. Again, temperature had the biggest effect on TBARS values, antioxidant concentration had the second largest effect, and packaging had the smallest effect. Most treatments were significantly different from each other, with the exception of two treatments where packaging was not significantly different. Samples stored at 25°C had TBARS values ranging from 0.22 ng/ml MDA for samples containing 500 ppm vitamin E to 0.38 ng/ml MDA for control samples with no vitamin E. Samples stored at 50°C had TBARS values ranging from 0.41 ng/ml MDA for samples containing 500 ppm vitamin E to 0.77 ng/ml MDA for control samples with no vitamin E. Samples stored at 50°C with 200 ppm and 500 ppm vitamin E concentrations showed no significant differences between the plastic and metallized packaging materials. Samples with 500 ppm vitamin E stored at 25°C had the lowest TBARS values.

Figure 8 shows TBARS values for all treatments at week 23. Again, temperature had the greatest effect on TBARS values. Samples stored at 25°C had values ranging from 0.47 ng/ml MDA in samples containing 500 ppm vitamin E to 0.65 ng/ml MDA for control samples with no vitamin E. Samples stored at 50°C had TBARS values ranging from 0.88 ng/ml MDA in samples containing 500 ppm vitamin E to 1.14 ng/ml MDA for control samples with no vitamin E. All four samples with 0 ppm vitamin E had significantly different TBARS values for all treatments involving temperature and packaging (a, b, e, and f). The samples with 200 ppm and 500 ppm vitamin E also had significantly different TBARS values for samples stored at 25°C (g, h, i, and j). However, for samples with 200 ppm and 500 ppm vitamin E stored at 50°C, the TBARS values for three of the four treatments were not significantly different.

### 3.2. Temperature Effects on Rancidity

Storage temperature (25°C and 50°C) of samples had a significant effect on the development of oxidative rancidity. Peroxide values ranging from 23 to 27 meq/kg represent the early stages of rancidity and peroxide values greater than 30 indicate complete rancidity; and TBARS values greater than 0.6 ng/ml typically indicate the early stages of rancidity [18]. From Figures 1 and 2, the time to onset of rancidity from peroxide value data was 15 weeks at 25°C and 11 weeks at 50°C. From Figures 3 and 4, the time to onset of rancidity from TBARS data was 19 weeks at 25°C and 11 weeks at 50°C. It can be clearly observed that the peanut butter bites stored at lower temperature take longer to show signs of rancidity for both peroxide and TBARS values. Since the oxidation process is known to be affected by temperature, it is not surprising that the formation of both primary and secondary oxidation products is slower at lower temperatures.

### 3.3. Effect of Packaging Material on Oxidative Rancidity

PV and TBARS values for samples stored in the plastic and metallized packaging were similar for most treatments, and the differences were the smallest at high-temperature storage. Saran™ wrap was used as an initial packaging material to maintain the shape of the peanut butter bites, and the metallized and plastic packaging were used as a second packaging layer. Oxygen and moisture permeability values are critical parameters that determine the suitability of a particular packaging material. In determination of oxidative stability in high-lipid food systems like peanut butter, oxygen permeability of packaging material plays a more significant role than the moisture permeability. From the results, peanut butter bite samples stored in metallized packaging in combination with Saran™ were less oxidized than the samples stored in plastic packaging in combination with Saran™. These results can be expected due to the lower oxygen permeability in the metallized packaging material compared to that of the plastic material (see Table 2). Also, the use of the Saran™ as a primary packaging could have minimized differences between the two types of packaging.

### 3.4. Effect of Antioxidant Concentration on Oxidative Rancidity

To delay the oxidative rancidity, three different natural antioxidants: vitamin A, beta-carotene, and vitamin E, were incorporated into the formulation and lipid oxidation was analyzed during a preliminary experiment. Out of 3 antioxidants tested, vitamin E was found to be the most effective in delaying the lipid oxidation of peanut butter bites. Hence, vitamin E was chosen for use in this study, using 3 different concentrations of vitamin E: 0 ppm, 200 ppm, and 500 ppm. A previous study involving antioxidants in butter found *α*-tocopherol to be an effective natural antioxidant [6].

From the peroxide value and TBARS assays, different levels of added vitamin E had a significant effect on oxidation at each time interval. From week 0 to week 23, there was an overall significant effect of antioxidant concentration on the oxidative rancidity. Vitamin E is utilized for its antioxidant properties and provides protection against autoxidation, but as time proceeds, it becomes depleted. In samples with the highest level of vitamin E (500 ppm), the onset of rancidity occurs during week 15 for samples stored at 25°C, where the PV ranged from 23 to 27 meq/kg. For samples with lower levels of vitamin E (0 or 200 ppm), the onset of rancidity occurs several weeks earlier based on the PV values. From the TBARS assay, the onset of rancidity (TBARS value > 0.6 ng/ml) occurred during week 15 for samples containing 500 ppm vitamin E stored at 50°C. For the samples containing 200 ppm vitamin E stored at 50^o^ C, the onset of rancidity occurred during week 11, and for the samples containing no vitamin E the onset of rancidity occurred at 3 weeks. The positive effect of the antioxidant was clear, and the antioxidant activity seemed to be concentration dependent.

### 3.5. Oxidation Rate

Oxidation rate based on changes in peroxide value was calculated for three different time intervals: 0-2 weeks, 2-5 weeks, and 5-23 weeks, and is shown in Table 3. For all 12 treatments, the rate of oxidation is highest during the first 2 weeks of storage, ranging from 3.80 to 5.05 meq/kg-week. Gradually, the slope decreases from week 2 to week 5 with a range of 1.73 to 2.62 meq/kg-week, indicating a decrease in oxidation rate during the intermediate storage period compared to the initial rate. During week 5 to week 23, the oxidation rate was at a minimum, ranging from 0.51 to 0.61 meq/kg-week. Oxidation rates were higher for samples stored at 50°C than those stored at 25°C, but the differences are more pronounced at the early time interval (0-2 weeks) and seem to level out as storage time progresses.

Table 4 shows the oxidation rates based on TBARS values for the 12 treatments. For TBARS values, rates were calculated at four different time intervals: 0-1 week, 1-2 weeks, 2-5 weeks, and 5-23 weeks. The additional time interval was included because the changes between weeks one and two were not consistent. For samples stored at 50°C, all treatments had highest oxidation rates during the first week, with lower rates as time progressed. For samples stored at 25°C, there was less difference between oxidation rates during weeks one and two, and in some cases, week two had a higher rate. During the first week, rates ranged from 0.478 to 0.148 ng/ml-week at 50°C storage, and from 0.112 to 0.033 ng/ml-week at 25°C storage. The highest oxidation rates were seen in samples with no vitamin E. During week 2, all the rates are more uniform, ranging from 0.031 to 0.146 ng/ml-week. The rates decreased as time progressed, and rates from week 2 to week 5 had a range of 0.027-0.061 ng/ml-week. During week 5 to week 23, the oxidation rate was at a minimum, ranging from 0.015 to 0.026 ng/ml-week.

### 3.6. Statistical Analysis

Statistical analyses were performed to identify significant differences among the rates of oxidation of peanut butter bites due to the effects of temperature, packaging, antioxidant concentration, and different time intervals. From the ‘type 3 tests of fixed effects', all four source effects and all possible interactions were tested using the *F*-test at 95% confidence interval. Results showed that for both PV and TBARS assays, overall source effects and all possible interactions were significant (*p* < 0.05). It is apparent that there are significant interactions between temperature, packaging, antioxidant concentration, and time interval.

## 4. Conclusions

This study provides insights on oxidative stability of peanut butter bites and the effects of storage conditions such as storage temperature, packaging material, and concentration of antioxidant in the snack.

The major conclusions drawn from this study are as follows:

1. Storage temperature had a significant effect on oxidative rancidity of peanut butter bites. The 50°C storage temperature resulted in oxidation values that were nearly 100% higher than those observed at 25°C storage
2. Addition of the antioxidant vitamin E was found to be effective in delaying the oxidative rancidity of peanut butter bites, and antioxidant level had a significant effect on both peroxide and TBARS values
3. Overall, the effects of packaging on oxidative rancidity were significant, with the metallized package resulting in the best oxidative barrier. However, differences between the two types of packaging were not significant for several treatments at specific time points
4. Time to onset of rancidity varied from 11 weeks to 15 weeks, depending on the treatment. The shortest times occurred at high-temperature storage
5. Overall, temperature, packaging, antioxidant concentration, and time interval had a significant effect on oxidation
6. The slowest oxidation rates occurred in samples prepared with 500 ppm vitamin E, in the metallized packaging material, and stored at 25°C

## Figures and Tables

**Table 1:** Formulation of peanut butter bites with different levels of vitamin E.

Ingredient	Formula 1 (g)	Formula 2 (g)	Formula 3 (g)
Peanut butter	39.50	39.50	39.50
Peanut flour	2.00	2.00	2.00
Pea protein	2.00	2.00	2.00
Cacao butter	1.00	1.00	1.00
Mono-di-glycerides	1.00	1.00	1.00
Gellan gum	0.04	0.04	0.04
Brown sugar	3.00	3.00	3.00
Water	0.96	0.96	0.96
Enzyme TG-GS	0.50	0.50	0.50
Vitamin E	0.00 (0 ppm)	0.01 (200 ppm)	0.025 (500 ppm)
Total	50 g	50.01 g	50.025 g

**Table 2:** Characteristic properties of packaging materials.

Packaging	Level	Material	Moisture permeability (10^−5^ g/in^2^-hr)	Oxygen permeability (10^−5^ cc/in^2^-hr)
Saran™ wrap	Primary	Polyethylene	8528.45553	3.53828
Metallized pouch	Secondary	Polyester and polyethylene bonded film	4.1875	0.33332
Plastic pouch	Secondary	Polyethylene	776.671	0.49275

**Table 3:** Rate of oxidation in meq/kg-week for peroxide values.

Antioxidant	Storage combination	Oxidation rate 0-2 weeks	Oxidation rate 2-5 weeks	Oxidation rate 5-23 weeks
0 ppm Vit E	Plastic 25 C	4.08	2.15	0.59
	Plastic 50 C	5.05	2.53	0.53
	Metallized 25 C	3.98	2.17	0.54
	Metallized 50 C	5.00	2.50	0.51

200 ppm Vit E	Plastic 25 C	3.95	1.95	0.55
	Plastic 50 C	4.80	2.62	0.52
	Metallized 25 C	4.00	1.73	0.57
	Metallized 50 C	4.63	2.58	0.57

500 ppm Vit E	Plastic 25 C	3.90	2.08	0.60
	Plastic 50 C	4.55	2.58	0.56
	Metallized 25 C	3.80	2.08	0.58
	Metallized 50 C	4.38	2.60	0.61

Range		3.80-5.05	1.73-2.62	0.51-0.61

**Table 4:** Rate of oxidation in ng/ml-week for TBARS values.

Antioxidant	Storage combination	Oxidation rate 0-1 weeks	Oxidation rate 1-2 weeks	Oxidation rate 2-5 weeks	Oxidation rate 5-23 weeks
0 ppm Vit E	Plastic 25 C	0.107	0.106	0.032	0.015
	Plastic 50 C	0.477	0.042	0.061	0.021
	Metallized 25 C	0.112	0.099	0.030	0.018
	Metallized 50 C	0.478	0.031	0.048	0.016

200 ppm Vit E	Plastic 25 C	0.065	0.106	0.027	0.017
	Plastic 50 C	0.256	0.040	0.049	0.020
	Metallized 25 C	0.066	0.081	0.030	0.019
	Metallized 50 C	0.240	0.045	0.054	0.022

500 ppm Vit E	Plastic 25 C	0.033	0.085	0.032	0.018
	Plastic 50 C	0.154	0.052	0.057	0.024
	Metallized 25 C	0.034	0.031	0.024	0.014
	Metallized 50 C	0.148	0.146	0.030	0.026

Range		0.033-0.478	0.031-0.146	0.027-0.061	0.015-0.026

## Data Availability

All data collected in support of this study are available from the corresponding author upon request.
